# Remarkable Response to Chemo-immunotherapy In Anaplastic Thyroid Cancer

**DOI:** 10.1210/jcemcr/luaf160

**Published:** 2025-07-28

**Authors:** Francesca Carosi, Maria Concetta Nigro, Giambattista Siepe, Andrea Repaci, Laura Deborah Locati, Margherita Nannini

**Affiliations:** Department of Medical and Surgical Sciences, University of Bologna, Bologna 40138, Italy; Department of Medical and Surgical Sciences, University of Bologna, Bologna 40138, Italy; Radiation Oncology Unit, IRCCS Azienda Ospedaliero-Universitaria di Bologna, Bologna 40138, Italy; Division of Endocrinology and Diabetes Prevention and Care, IRCCS Azienda Ospedaliero-Universitaria di Bologna, Bologna 40138, Italy; Internal Medicine and Therapeutics Department, University of Pavia, Pavia 27100, Italy; Medical Oncology Unit, Istituti Clinici Scientifici Maugeri IRCCS, Pavia 27100, Italy; Department of Medical and Surgical Sciences, University of Bologna, Bologna 40138, Italy; Medical Oncology Unit, IRCCS Azienda Ospedaliero-Universitaria di Bologna, Bologna 40138, Italy

**Keywords:** anaplastic thyroid cancer, immunotherapy, chemotherapy, multimodal treatment

## Abstract

Anaplastic thyroid cancer (ATC) is an aggressive and lethal malignancy with limited therapeutic options and poor prognosis. In recent years, the therapeutic arsenal of locally advanced or metastatic ATC has been expanded, with V-Raf murine sarcoma viral oncogene homolog B (*BRAF)/*mitogen-activated protein kinase kinase-MAPKK *(MEK)* inhibitors for the subset of *BRAF V600E*-mutant ATC. For *BRAF* wild-type ATC and without other actionable alterations, the most promising strategy is certainly immune checkpoint inhibitors, which have shown activities both in monotherapy or in combination regimens. However, access to novel treatments is heterogeneous worldwide for ATC patients, and activity of immunotherapy as a single agent is limited.

We report the case of a patient with locally advanced *BRAF* wild-type ATC who achieved a near-complete and durable response following a multimodal treatment approach combining chemotherapy (carboplatin and paclitaxel), immunotherapy (pembrolizumab), and external beam radiotherapy. Pembrolizumab monotherapy was continued as maintenance therapy, and disease control was maintained for over 1 year.

This case highlights the potential efficacy of chemo-immunotherapy in *BRAF* wild-type ATC, especially when a rapid tumor reduction is required. It supports the use of immune checkpoint inhibitors combined with cytotoxic agents as a viable therapeutic option in this aggressive tumor subtype.

## Introduction

Anaplastic thyroid carcinoma (ATC) is a rare, undifferentiated, and highly aggressive malignancy, regarded as 1 of the deadliest human cancers. It carries a dismal prognosis, with a median overall survival (OS) of approximately 4 months and a 6-month OS rate of only 35% from the time of diagnosis [1]. ATC may occur de novo or arise from preexisting differentiated thyroid cancer. It is characterized by a high mutational burden, marked genomic instability, and dysregulated angiogenesis, all of which contribute to its rapid progression and resistance to therapy [2]. Histologically, ATC displays a high mitotic index, frequent lymphovascular invasion, and marked cellular dedifferentiation. At diagnosis, nearly 50% of cases are deemed inoperable due to bulky primary tumors, extensive extrathyroidal extension, or the presence of locoregional and distant metastases.

To date, the standard treatment for unresectable or metastatic ATC has largely relied on cytotoxic chemotherapy, typically involving paclitaxel or doxorubicin-based regimens. However, these approaches have yielded limited survival benefits and minimal improvement in quality of life [3]. Advances in molecular profiling have recently expanded the therapeutic landscape for ATC, enabling the development of targeted therapies. In particular, the combination of the *BRAF* inhibitor dabrafenib and the *MEK* inhibitor trametinib is now the standard of care for patients with *BRAF V600E*-mutant locally advanced or metastatic ATC. This regimen has demonstrated an objective response rate (ORR) of 56%, with 12-month progression-free survival and OS rates of 43.2% and 51.7%, respectively [4].

For patients with *BRAF* wild-type tumors and no other actionable alterations, immune checkpoint inhibitors (ICIs) have emerged as a promising treatment option. ICIs have shown antitumor activity both as monotherapy and in combination regimens, based on data from clinical trials and retrospective series (Table 1). However, access to these therapies remains inconsistent worldwide, and the rapid progression of ATC often outpaces regulatory approval processes and enrollment in clinical trials [5]. Additionally, due to the aggressive nature of the disease and its frequent involvement of vital structures, many patients experience clinical deterioration before treatment can be initiated.

**Table 1.** Currently published clinical trials and retrospective series on immunotherapy in anaplastic thyroid cancer

Author, year	Design	Drug	Line	No. patients	ORR	Other endpoints
Capdevila et al, 2020 [6]	Phase I/II	Spartalizumab 400 mg q4w	First or second line and beyond	42	19% [7.1% CR (3 pts), 11.9% PR (5 pts)]	1-year survival 52.1% in PD-L1 positive pts
Hatashima et al, 2022 [7]	Case series	Pembrolizumab or nivolumab	Any line	13	16% [15.3% PR (2 pts), 23% SD (3 pts)]	mPFS 1.9 mo mOS 4.4 mo
Lorch et al, 2020 [8]	Phase II	Nivolumab 3 mg/kg q2w + ipilimumab 1 mg/kg q6w	Second line and beyond	10	30% [30% PR (2 pts)]	—
Cabanillas et al, 2024 [9]	Phase II	Atezolizumab + vemurafenib/cobimetinib, cobimetinib, or bevacizumab	Second line and beyond	42	31% cohort 1 (BRAF): 50%; cohort 2 (MEK): 14%; cohort 3 (VEGF): 33%.	mOS 18.23 mo mPFS 7.98 mo
Chintakuntlawar et al, 2019 [10]	Phase II	Pembrolizumab 200 mg q3w + chemoradiotherapy (docetaxel/doxorubicin 20 mg/mq weekly)	First line	3	All with early locoregional tumor response, but all died < 6 mo (study closed)	—
Song et al, 2024 [11]	Retrospective series	Dabrafenib/trametinib, lenvatinib, or anlotinib + pembrolizumab, sintilimab, or camrelizumab	First line	18	61% [27.7% CR (5 pts), 33.3% PR (6 pts)]	mOS 14 mo (better in BRAF V600E mut ATC) 12-mo survival rate 55.6%; 38.9% underwent surgical resection
Dierks et al, 2021 [12]	Retrospective series	Lenvatinib (14-24 mg daily) + pembrolizumab 200 mg q3w up to 40 mo	Second line and beyond	6	66% [66% CR (4 pts), 16% SD (1 pts), 16% PD (1 pts)]	mPFS 16.5 mo mOS 18.5 mo (3 ATC pts still alive without relapse)
Soll et al, 2024 [13]	Retrospective series	Lenvatinib (14-24 mg daily) + pembrolizumab 200 mg q3w up to 40 mo	First line	5	20% [20% PR (1 pts), 60% SD (3), 20% NA (1 pts, formally NA for concomitant infectious thyroiditis, SD for more than 1 year)]	mPFS 4.7 mo mOS 6.3 mo
Dierks et al, 2020 [14]	Phase II (ATLEP trial)	Lenvatinib (20 mg daily) + pembrolizumab 200 mg q3w up to 2 years	Second line and beyond	27	34.3% [51.9% PR (14/27), 48.1% SD (13/27)]	CBR 96.3% mPFS 9.5 mo mOS 10.25 mo; 25% have long-term responses (more than 2 years)
Hamidi et al, 2024 [21]	Retrospective series	DT (dabrafenib/trametinib), DTP (pembrolizumab added upfront or at PD), neoadjuvant (DT before surgery, pembrolizumab added after surgery)	First line	71 (48 treated with DTP)	64.5% [DT], 73.3% [upfront DTP]	mOS 17 mo mPFS 11 mo (23% pts in the neoadjuvant group)
Iyer et al, 2018 [15]	Retrospective series	Pembrolizumab + TKI (lenvatinib, dabrafenib/trametinib, trametinib alone)	Salvage pembrolizumab after PD to TKI	12	40% [40% PR (5 pts), 33% SD (4 pts), 25% PD (3 pts)]	mOS 6.93 mo mPFS 2.96 mo
Sehgal et al, 2024 [16]	Phase II	Nivolumab 3 mg/kg q2w and ipilimumab 1 mg/kg q6w up to 2 years	Second line and beyond	10	30.0% [3/10 pts]	CBR 50%

Abbreviations: CBR, clinical benefit rate; CR, complete response; mo, months; mOS, median overall survival; mPFS, median progression-free survival; ORR, objective response rate; PD, progressive disease; PR, partial response; pts, patients; SD, stable disease; TKI, tyrosine kinase inhibitors.

Herein we present the case of a patient with locally advanced, *BRAF* wild-type ATC who was treated with a sequential multimodal regimen including chemo-immunotherapy (carboplatin, paclitaxel, and pembrolizumab) followed by external beam radiotherapy and maintenance pembrolizumab. This approach resulted in a sustained near-complete radiologic response lasting over 1 year.

## Case Presentation

The patient was a 59-year-old woman who, in December 2018, was incidentally found to have a 26 × 15 mm thyroid nodule in the left lobe, characterized by regular margins and a predominantly cystic composition with some solid components and colloid-hemorrhagic content. The lesion was classified as EU-TIRADS 3 according to the European Thyroid Imaging Reporting and Data System and was deemed benign (Bethesda category II) based on fine-needle aspiration cytology.

**Figure 1.**
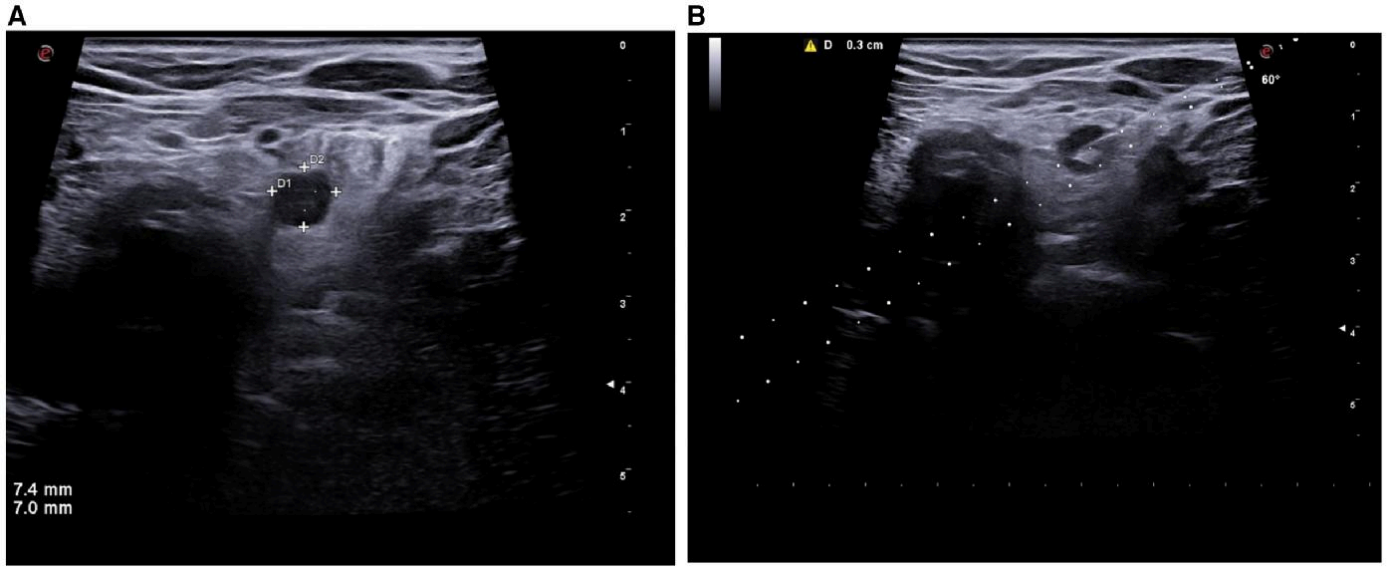
(A-B) Ultrasound image of voluminous nodule of the left thyroid lobe with regular margins and mixed solid and predominantly cystic component with colloid-hematic content.

In July 2023, she presented with progressive dysphagia to both solids and liquids, along with a sensation of cervical compression. A repeat thyroid ultrasound revealed a markedly enlarged nodule in the left thyroid lobe, measuring 40.2 × 36 × 61.8 mm, with similar features to the original lesion—mixed solid and predominantly cystic morphology, regular margins, and colloid-hemorrhagic (Fig. 1A-1B). A second fine-needle aspiration revealed a sparse cluster of atypical cells with enlarged nuclei and prominent nucleoli, consistent with Bethesda category V cytology. Additionally, an abnormal lateral cervical lymph node at level VI measuring 7.4 × 7.0 × 9.1 mm was detected.

In October 2023, the patient underwent a left hemithyroidectomy. A total thyroidectomy was not initially performed due to the absence of clinical or cytological suspicion of malignancy at that time. Histopathologic examination of the surgical specimen revealed multiple fragments of squamous cell carcinoma exhibiting diffuse immunohistochemical positivity for p40, PAX-8, CK7, and CK19, consistent with a diagnosis of ATC with squamous differentiation. Programmed death-ligand 1 (PD-L1) testing showed a high combined positive score of 85%. Comprehensive genomic profiling by next-generation sequencing, using both a custom laboratory-developed multigene panel for mutation analysis and the Oncomine Focus Assay for gene fusion detection, identified a *TP53* mutation (p.Trp53Ter, c.158G > A, Exon 4). No alterations were detected in *BRAF*, *RET*/*PTC*, *NTRK*, or *ALK* genes.

**Figure 2.**
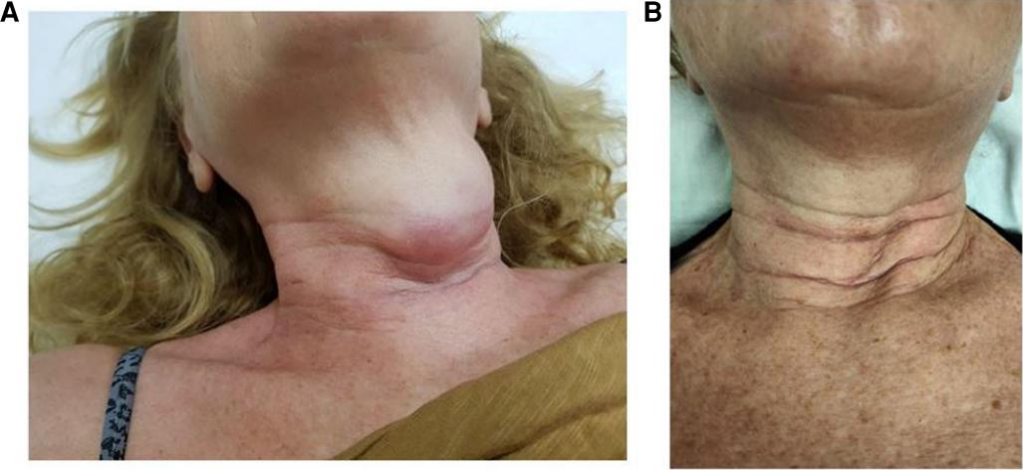
**(**A) Clinical evaluation of a voluminous local relapse/persistence of ATC. (B) Rapid remarkable clinical tumor response in an ATC patient treated with chemo-immunotherapy combined with radiotherapy. Abbreviation: ATC, anaplastic thyroid cancer.

One month after surgery, physical examination revealed a painful, firm, and indurated mass near the surgical scar, with overlying erythematous skin suggestive of lymphangitic involvement. A palpable right lateral cervical lymph node at level V, approximately 2 cm in size, was also noted (Fig. 2A). At this stage, the patient exhibited clear clinical evidence of locoregional recurrence and was symptomatic. Surgical intervention was deemed unfeasible due to the extent and progression of disease.

## Diagnostic Assessment

**Figure 3.**
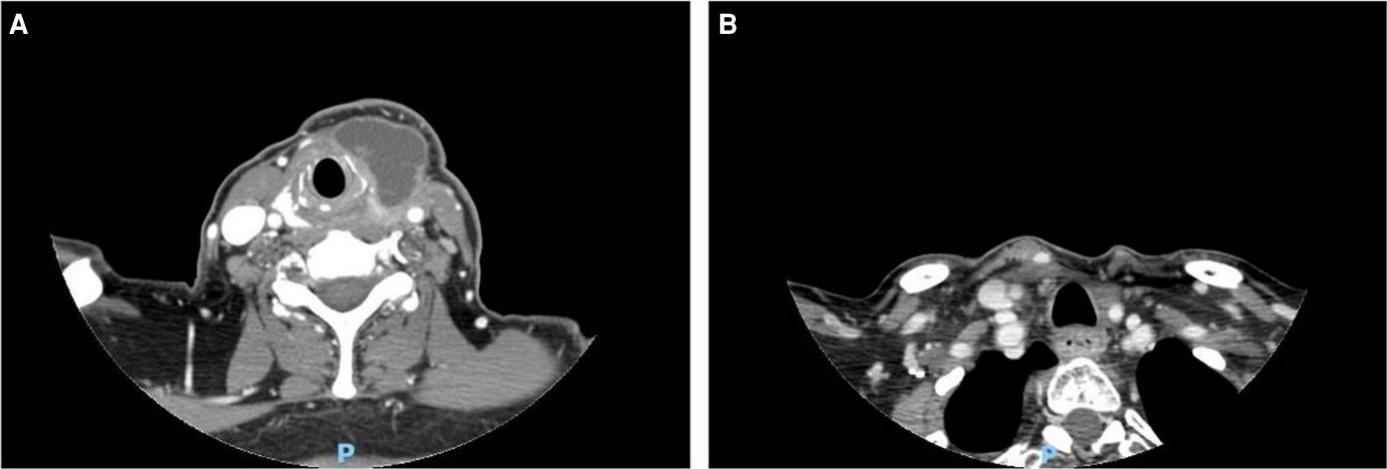
(A) Axial CT scan demonstrating a locoregional relapse of ATC: a large cystic formation at paratracheal site, displacing the left submandibular gland and infiltrating trachea, bordering muscles and skin. (B) Axial CT scan of a locoregional relapse of ATC treated with chemo-immunotherapy combined with radiotherapy, demonstrating a nearly complete radiological response. Abbreviations: ATC, anaplastic thyroid cancer; CT, computed tomography.

A total body computed tomography (CT) scan performed in November 2023, following surgery, revealed a large cystic lesion measuring 45 × 39 × 24 mm located in the left paratracheal space. The mass displaced the left submandibular gland and demonstrated evidence of tracheal invasion, with close proximity to the adjacent musculature and overlying skin. Additionally, a necrotic lymph node was identified in the left paratracheal region, and multiple pathological subcutaneous lymph nodes were observed beneath the right sternocleidomastoid muscle (Fig. 3A). Fluorine-18 fluorodeoxyglucose positron emission tomography showed intense hypermetabolic activity at the left thyroid bed, with a maximum standardized uptake value (SUVmax) of 34.8, consistent with residual disease. Additional focal areas of increased glucose uptake were noted in paratracheal lymph nodes at left level VI (SUVmax 14.1) and in a left supraclavicular lymph node (SUVmax 5.7), further supporting the presence of locoregional disease recurrence.

## Treatment

Given the rapid progression of the residual disease in close proximity to vital structures and the patient's symptomatic clinical status, systemic treatment was promptly initiated. First-line therapy consisted of a 3-weekly regimen of carboplatin dosed at area under the curve 5, paclitaxel at 175 mg/m², and the anti-PD-1 immune checkpoint inhibitor pembrolizumab at 200 mg every 21 days. Pembrolizumab was introduced from the second cycle under an off-label compassionate use protocol.

Between November 2023 and January 2024, the patient completed 3 cycles of chemo-immunotherapy. Treatment-related toxicities, assessed according to the Common Terminology Criteria for Adverse Events, included grade 4 neutropenia, grade 2 anemia, and grade 1 thrombocytopenia, which were clinically manageable.

## Outcome and Follow-up

During the course of treatment, a rapid and substantial clinical tumor response was observed following 3 cycles of chemotherapy, 2 of which were administered in combination with pembrolizumab (Fig. 2B). A contrast-enhanced CT scan performed in February 2024 demonstrated a near-complete radiological response of the locoregional tumor relapse, along with significant reduction in the size of pathological lateral cervical lymph nodes (Fig. 3B).

**Figure 4.**
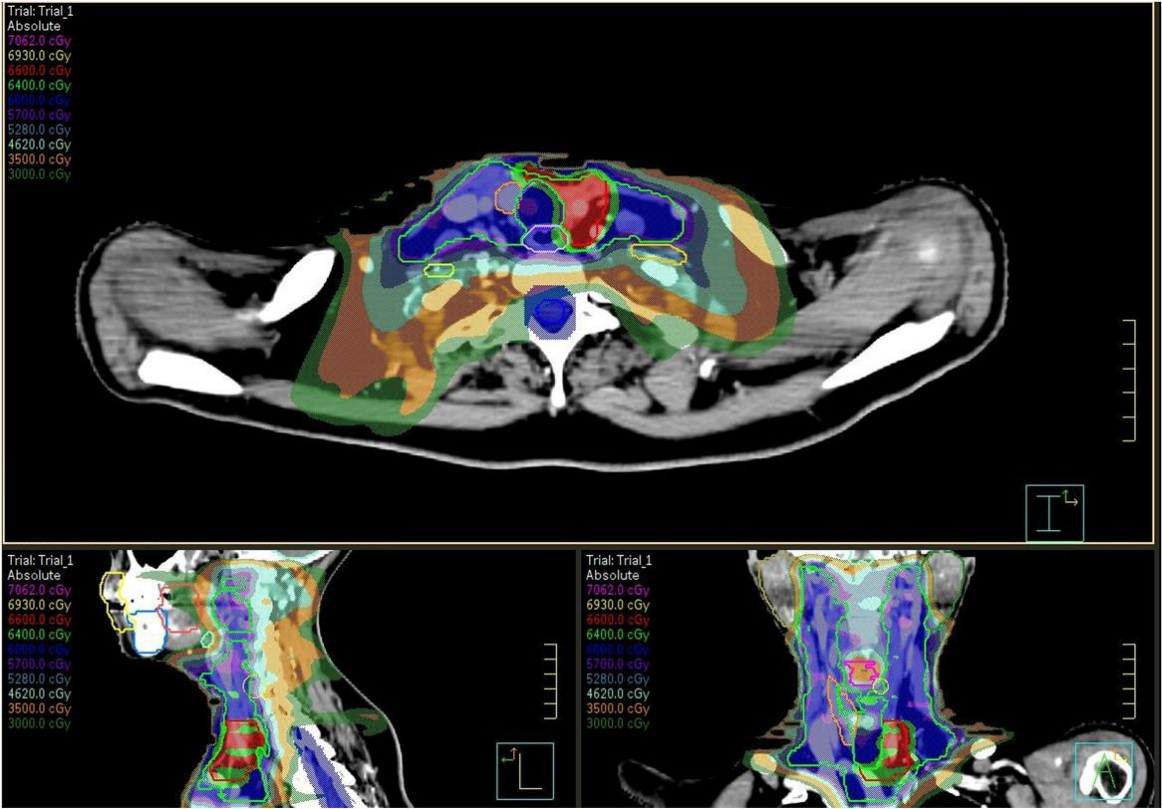
Simultaneous integrated boost volumetric-modulated arc radiotherapy.

Between March and May 2024, the patient underwent consolidative locoregional external beam radiotherapy. A total dose of 66 Gy in 30 fractions was delivered to the primary site of relapse, while 60 Gy was administered to the bilateral lateral cervical lymph node chains (levels II-VI) using a simultaneous integrated boost volumetric-modulated arc therapy technique (Fig. 4).

Given the radiological and clinical stability achieved, maintenance monotherapy with pembrolizumab was initiated in July 2024 and continued for an additional 12 cycles. A follow-up whole-body CT scan performed in October 2024 confirmed stable disease, and the patient has since continued on pembrolizumab maintenance at a flat dose of 400 mg every 6 weeks, which remains ongoing at the time of this report.

## Discussion

ATC remains 1 of the most aggressive and lethal solid tumors, with median survival measured in months and historically limited therapeutic options. Conventional cytotoxic chemotherapy (including taxanes, doxorubicin, and platinum-based agents) has demonstrated poor efficacy, with ORRs of only 15% to 25% and no meaningful improvement in OS [17]. In recent years, however, the therapeutic landscape of advanced ATC has evolved considerably. The introduction of targeted therapies, particularly the *BRAF* inhibitor dabrafenib in combination with the *MEK* inhibitor trametinib, has significantly improved outcomes in patients with *BRAF V600E*-mutant ATC, achieving ORRs of 56% and durable responses in a substantial proportion of cases [4]. For patients with *BRAF* wild-type tumors or those lacking other actionable genomic alterations, ICIs have emerged as a promising alternative. ICIs, administered either as monotherapy or in combination with other agents such as tyrosine kinase inhibitors or dual ICI regimens, have shown ORRs ranging from 16% to 19% for single agents to as high as 66% for combinations like pembrolizumab plus lenvatinib in retrospective series [12].

ATC is considered highly immunogenic compared to other thyroid cancers, and responses to immunotherapy have been reported regardless of PD-L1 expression status [6, 18]. The combination of pembrolizumab and lenvatinib appears particularly promising due to the immunomodulatory effects of antiangiogenic therapy on the tumor microenvironment, including enhancement of T-cell infiltration and reversal of immune suppression, mechanisms supported by both preclinical and clinical studies. An ORR of 66% was reported in a retrospective series of 6 ATC with this combination. However, access to this treatment strategy remains limited and is not yet standardized across clinical practice worldwide [19]. Moreover, combining lenvatinib with ICIs may help to overcome both primary and acquired resistance to immunotherapy [20]. Furthermore, emerging data suggest that pembrolizumab may enhance the efficacy of targeted therapies such as dabrafenib and trametinib in *BRAF V600E* mutant ATC, with an impressive impact on overall survival [21].

Beyond systemic combinations, the integration of radiotherapy with immunotherapy has also demonstrated encouraging results in ATC. Although the underlying immune mechanisms are still under investigation, several reports have described a potentiating or “abscopal” effect, defined as regression of nonirradiated tumor sites following localized radiotherapy in the context of immune activation [22-25].

Importantly, the immunomodulatory properties of cytotoxic chemotherapy should not be overlooked. Agents such as carboplatin and taxanes can contribute to antitumor immunity by promoting immunogenic cell death, enhancing tumor antigen presentation, increasing tumor-infiltrating lymphocytes, and reducing immunosuppressive cell populations. For instance, carboplatin has been shown to upregulate PD-1 mRNA expression, while taxanes may facilitate immune cell recruitment and activation within the tumor microenvironment [26].

In this context, we report the case of a patient with locally advanced *BRAF* wild-type ATC who achieved a near-complete and durable radiologic response following sequential chemo-immunotherapy (carboplatin, paclitaxel, and pembrolizumab), external beam radiotherapy, and maintenance pembrolizumab monotherapy. The observed tumor regression was both rapid and profound, especially when compared to expected outcomes from chemotherapy or immunotherapy alone [17], suggesting a synergistic or priming effect of the chemo-immunotherapy combination.

Given the aggressive clinical course of ATC and the frequent need for rapid disease control, a multimodal treatment approach that integrates chemotherapy, immunotherapy, and radiotherapy appears to be the most rational and effective strategy. Our case underscores the potential value of chemo-immunotherapy, particularly in patients lacking targetable mutations, for whom timely and substantial tumor shrinkage is essential to improve both prognosis and quality of life.

## Learning Points

- The standard of care for ATC has not yet achieved satisfactory results in terms of survival outcomes.
- For BRAF wild-type ATC without other targetable mutations, ICIs, both in monotherapy and in combined regimens, emerge as a novel therapeutic option.
- Chemo-immunotherapy could be a viable option as first-line treatment in ATC without actionable gene alterations, especially when achieving a rapid tumor shrinkage is clinically essential.

## Data Availability

**Original data generated and analyzed during this study are included in this published article.**
